# Liproxstatin-1 attenuates unilateral ureteral obstruction-induced renal fibrosis by inhibiting renal tubular epithelial cells ferroptosis

**DOI:** 10.1038/s41419-021-04137-1

**Published:** 2021-09-11

**Authors:** Bo Zhang, Xiang Chen, Feng Ru, Yu Gan, Bingsheng Li, Weiping Xia, Guoyu Dai, Yao He, Zhi Chen

**Affiliations:** Department of Urology, Xiangya Hospital, Central South University, Changsha, Hunan People’s Republic of China

**Keywords:** Cell death, RNAi, Urinary tract obstruction

## Abstract

Renal fibrosis is a common pathological process that occurs with diverse etiologies in chronic kidney disease. However, its regulatory mechanisms have not yet been fully elucidated. Ferroptosis is a form of non-apoptotic regulated cell death driven by iron-dependent lipid peroxidation. It is currently unknown whether ferroptosis is initiated during unilateral ureteral obstruction (UUO)-induced renal fibrosis and its role has not been determined. In this study, we demonstrated that ureteral obstruction induced ferroptosis in renal tubular epithelial cells (TECs) in vivo. The ferroptosis inhibitor liproxstatin-1 (Lip-1) reduced iron deposition, cell death, lipid peroxidation, and inhibited the downregulation of GPX4 expression induced by UUO, ultimately inhibiting ferroptosis in TECs. We found that Lip-1 significantly attenuated UUO-induced morphological and pathological changes and collagen deposition of renal fibrosis in mice. In addition, Lip-1 attenuated the expression of profibrotic factors in the UUO model. In vitro, we used RSL3 treatment and knocked down of GPX4 level by RNAi in HK2 cells to induce ferroptosis. Our results indicated HK2 cells secreted various profibrotic factors during ferroptosis. Lip-1 was able to inhibit ferroptosis and thereby inhibit the secretion of the profibrotic factors during the process. Incubation of kidney fibroblasts with culture medium from RSL3-induced HK2 cells promoted fibroblast proliferation and activation, whereas Lip-1 impeded the profibrotic effects. Our study found that Lip-1 may relieve renal fibrosis by inhibiting ferroptosis in TECs. Mechanistically, Lip-1 could reduce the activation of surrounding fibroblasts by inhibiting the paracrine of profibrotic factors in HK2 cells. Lip-1 may potentially be used as a therapeutic approach for the treatment of UUO-induced renal fibrosis.

## Introduction

Renal fibrosis is a common pathological condition of nearly all chronic and progressive renal diseases. It is characterized by the destruction of normal kidney structure, fibroblast proliferation, and excessive deposition of extracellular matrix (ECM) [1, 2]. Unilateral ureteral obstruction (UUO) is used as a model to induce renal fibrosis with the primary tubular injury due to obstructed urine flow [3]. Renal tubules are the major component of the kidney and the main sites in response to a variety of injuries, including hypoxia, proteinuria, toxins, metabolic disorders, and senescence [4]. UUO-induced mechanical stretching, oxidative stress, and other factors cause renal damage and death of tubular epithelial cells (TECs) [5]. Increasing evidence shows that TECs-derived profibrotic factors, including transforming growth factor-β1 (TGF-β1), connective tissue growth factor (CTGF), platelet-derived growth factor (PDGF), are a driving force in the progression of renal fibrosis due to their roles in activating surrounding fibroblast [4, 6]. The damaged TECs switch to a secretory phenotype and release soluble factors, which are hypothesized to activate the surrounding fibroblasts via paracrine effects [7, 8]. However, the exact cellular mechanisms involved are not well understood. Defining the pathophysiology of renal TECs is important for being able to prevent and arrest the progression of renal fibrosis.

Ferroptosis is a form of non-apoptotic regulated cell death, which is regulated by the lipid-repair enzyme glutathione peroxidase 4 (GPX4) and is driven by severe lipid peroxidation, which relies on the generation of reactive oxygen species (ROS) and iron overload [9]. Although the physiological function of ferroptosis remains largely unclear, its involvement in multiple human diseases has been confirmed. For instance, ferroptosis plays a significant role in multiple organ diseases, including brain, heart, kidney, and liver diseases [9–13]. Recent studies have demonstrated that ferroptosis plays an important role in ischemia/reperfusion-induced acute kidney injury (AKI) and is also a pathogenic factor in other AKI models [12, 14]. Accordingly, ferroptosis is a promising therapeutic target, especially in diseases dominated by kidney tubular necrosis [15]. Although AKI and chronic kidney disease (CKD) are interconnected, it is unknown whether ferroptosis occurs during renal fibrosis and its role has not been fully studied. Therefore, we were interested in exploring whether ferroptosis occurs during UUO-induced renal fibrosis.

Liproxstatin-1 (Lip-1) is a potent inhibitor of ferroptosis and can protect against ferroptosis-inducing agents, such as buthionine sulfoxamine (BSO), erastin, and (1S,3R)-RSL3 (RSL3). Moreover, Lip-1 does not interfere with other classical types of cell death, such as TNFα-induced apoptosis and H_2_O_2_-induced necrosis [14]. In recent years, Lip-1 has attracted the attention of researchers as it exhibits various kinds of pharmacological activities. Feng et al. found that Lip-1 can protect mouse myocardium against ischemia/reperfusion injury by decreasing voltage-dependent anion-selective channel 1 levels and restoring GPX4 levels [16]. Chen et al. reported that Lip-1 alleviates iron overload and attenuates morphine tolerance by increasing GPX4 levels, while reducing levels of malondialdehyde and ROS [17]. Friedmann et al. found that Lip-1 prevents both RSL3-induced death of primary human renal proximal TECs and *GPX4* deletion-induced acute renal failure [14]. Thus, Lip-1 inhibits ferroptosis and promotes cell survival; however, the mechanisms underlying these actions remain unclear.

In summary, we demonstrated that ferroptosis occurred in TECs during UUO-induced renal fibrosis. The ferroptosis inhibitor Lip-1 attenuated UUO-induced renal fibrosis by inhibiting ferroptosis-mediated TECs death. Lip-1 was able to attenuate the expression of profibrotic factors in UUO-induced kidney fibrosis tissue. Moreover, Lip-1 alleviated RSL3-induced and GPX4 knockdown-induced ferroptosis as well as the expression of profibrotic factors in human kidney 2 (HK2) cells, thereby indirectly inhibiting the proliferation and activation of fibroblasts. This study is expected to provide insight into a new approach and new mechanism for the treatment of UUO-induced renal fibrosis.

## Materials and methods

### Animals

Specific-pathogen-free 8-week-old male C57BL/6 mice were used in all studies. All experimental animal procedures were approved by the ethics committee of the Central South University Science Research Center (Changsha, China) (No. 2020sydw0319). The mice were anesthetized by sodium pentobarbital (80 mg/kg; intraperitoneal injection) and all efforts were made to minimize animal suffering during and following surgery.

### Animal model

The surgical procedure was performed as described in our previous study [18]. Briefly, the left ureter was exposed and ligated with 4-0 silk sutures, and the ligatures were dissected to prevent retrograde urinary tract infections. The sham operation was executed in a similar manner, but without ureter ligation. Twenty-four C57BL/6 mice were randomly divided into the following four groups: (1) sham group, (2) Lip-1 (>98% purity; MW 340.85, MedChemExpress, USA) group, (3) UUO group, and (4) UUO + Lip-1 group. The mice in the Lip-1 and UUO + Lip-1 groups were intraperitoneally injected with 200 μL Lip-1 (10 mg/kg/d, dissolved in DMSO, and then diluted with 0.9% NaCl) for 14 consecutive days after surgery. The mice in the sham and UUO groups were intraperitoneally injected with equal volumes of 0.9% NaCl solution. After the last treatment, all animals were anesthetized with sodium pentobarbital and sacrificed. Blood was collected from the ophthalmic arteries by retro-orbital bleeding. Part of the left kidney was fixed in a 4% paraformaldehyde solution and the remaining portion was snap-frozen in liquid nitrogen and stored at −80 °C for further analysis.

### Cell culture

The HK2 cells were purchased from Procell Life Science & Technology Co., Ltd. (Wuhan, China). The cells were cultured in Dulbecco’s Modified Eagle Medium (DMEM; Gibco, China) supplemented with 10% fetal bovine serum (FBS; CellMax, Australia) and 1% penicillin/streptomycin in a humidified atmosphere of 5% CO_2_. Human kidney fibroblasts were also purchased from Procell Life Science & Technology Co. The fibroblasts were resuspended in DMEM/F-12 medium (Hyclone, USA) containing 10% FBS (CellMax, Australia) and 1% penicillin/streptomycin and cultured in a humidified atmosphere of 5% CO_2_.

HK2 cells were seeded into dishes and grown to 80–90% confluence. Cells were then treated with 0.5 μM Lip-1 and/or 1.0 μM RSL3 (MedChemExpress, USA) for 24 h. After treating cells with RSL3/Lip-1 for 24 h, we cultured the cells with a new medium for collecting factors for another 24 h. All the medium was collected and used for enzyme-linked immunosorbent assay (ELISA) analysis. The remaining HK2-conditioned medium (HK2-CM) was used in co-culture experiments with the fibroblasts.

### Lentivirus-mediated RNA interference (RNAi) knockdown of GPX4

The RNAi sequence for human GPX4 (GGATGAAGATCCAACCCAA) was identified by using the manufacturer’s RNAi Designer program, and the negative control construct (control RNAi) having no homology with the human genome was created by a scrambled sequence (TTCTCCGAACGTGTCACGT). The sequences were cloned into the pGCSIL-Green Fluorescent Protein (GFP) to generate lentivirus vectors (GeneChem, Shanghai, China).

### Infection HK2 cells with lentivirus

HK2 cells were seeded in a 96-well plate or 12-well plate. For infection, the lentiviruses were diluted in a complete medium containing HitransG A, culturing cells for 12 h, then replaced with fresh medium for another 24 h to infected cells. Afterward, HK2 cells were treated with or without Lip-1 for a further 24 h. HK2 cells were divided into groups: Control group (infected with neg-RNAi-LV), Lip-1 group (neg-RNAi-LV + 0.5 μM Lip-1), GPX4 RNAi group (GPX4 RNAi-LV), GPX4 RNAi + Lip-1 (GPX4 RNAi-LV + 0.5 μM Lip-1) group. We collected all the medium from HK2 cells to detect fibrotic factors by ELISA analysis, and collected cells to detect cell viability and iron concentrations.

### Renal function analysis

Blood was retro-orbitally collected from the ophthalmic artery and sent to the Department of Clinical Laboratory, Xiangya Hospital, Central South University for determination of serum creatinine (Scr) and blood urea nitrogen (BUN) levels by colorimetry assays (Beckman AU5800, USA).

### Perls’ staining

The renal tissue was dissected and part of the tissue specimens was fixed in 4% paraformaldehyde solution and then embedded in paraffin tissue sectioning. For Perls’ staining, the tissues were incubated in freshly prepared Perls’ solution (1:1, 5% potassium ferrocyanide and 5% HCl) for 1 h, followed by washing with phosphate-buffered saline (PBS). A second incubation was then performed in 0.5% diamine benzidine tetrahydrochloride. After rinsing, the sections were observed under a light microscope. The proportion of positive cells was quantified from random 200× fields of each tissue slice and analyzed using ImageJ.

### E-cadherin and terminal deoxynucleotidyl transferase (TdT)-mediated dUTP nick end labeling (TUNEL) co-staining

For the detection of TUNEL-positive cells, TUNEL apoptosis detection kit (Roche, USA) according to the instructions provided by the manufacture. To determine whether the cell death occurred mainly in renal TECs, the co-staining of E-cadherin (epithelial cell marker) with TUNEL were evaluated in kidney tissues using immunofluorescence staining. The number of TUNEL-positive nuclei per field was counted in four non-repeating micrographs for each sample.

### Determination of renal GSSG/GSH ratio

The ratio of oxidized glutathione (GSSG) to total glutathione (GSH, reduced GSH + GSSG) was determined by GSH and GSSG Assay Kit (Beyotime, China) according to the manufacturer’s instructions. The absorbance at 412 nm was measured by a microplate reader.

### Immunohistochemistry, immunofluorescence, and histopathological

Hematoxylin and eosin (H&E) staining, Masson’s trichrome staining, immunohistochemistry, and immunofluorescence were performed as described previously [18]. For immunohistochemical staining, the sections were incubated with primary antibodies against GPX4 (1:100; Affinity Biosciences, China), collagen I (1:100; Servicebio, China), TGF-β1 (1:100; Servicebio, China), CTGF (1:100; Servicebio, China), and PDGF (1:100; Servicebio, China) overnight at 4 °C. The sections were then washed and incubated at room temperature with a horseradish peroxidase (HRP)-conjugated secondary antibody (1:100; Sigma-Aldrich, USA). For immunofluorescence staining, slide-mounted tissues were blocked with 5% bovine serum albumin for 30 min and then incubated overnight at 4 °C with antibodies specific for E-cadherin (1:100; Sigma-Aldrich, USA), GPX4 (1:100; Affinity Biosciences, China), and alpha-smooth muscle actin (α-SMA, 1:100; Abcam, UK), followed by staining with corresponding secondary antibodies (1:200, Beyotime, China) at room temperature. Cell nuclei were stained with 4,6-diamidino-2-phenylindole (DAPI; Proteintech, China). Images were acquired using a microscope with a mounted camera (Nikon, Tokyo, Japan).

### Western blotting

Renal tissues, HK2 cells, and human kidney fibroblasts were lysed and ultrasonicated in RIPA lysis buffer (Beyotime Biotechnology, China) containing a protease inhibitor cocktail (Roche Diagnostics, Indianapolis, IN, USA). Total protein concentrations were measured using a BCA kit (Thermo Scientific, USA). Protein samples were separated by sodium dodecyl sulfate-polyacrylamide gel electrophoresis and electrotransferred to polyvinylidene fluoride membranes (Millipore, USA). After blocking in 5% fat-free milk for 2 h, the membranes were incubated with the primary antibodies specific for glyceraldehyde 3-phosphate dehydrogenase (GAPDH, 1:2000; Servicebio, China), collagen I (1:500; Servicebio, China), α-SMA (1:500; Servicebio, China), GPX4 (1:2000; Affinity Biosciences, China) overnight at 4 °C. The membranes were then incubated with HRP-conjugated secondary antibodies (1:5000 goat anti-rabbit IgG, Boster, or 1:3000 goat anti-mouse IgG, Millipore, USA) for 1 h at room temperature. Protein bands were detected using enhanced chemiluminescence reagents and a Molecular Imager ChemiDoc XRS System (Bio-Rad, USA). The abundance of the proteins was analyzed using Image Lab analysis software and normalized to GAPDH protein levels.

### ELISA analysis of TGF-β1, CTGF, and PDGF

ELISA was used to determine TGF-β1, CTGF, and PDGF levels released from HK2 cells. Clarified supernatants of HK2-CM were assayed using ELISA kits specific for human TGF-β1, CTGF, and PDGF [Multisciences (Lianke) Biotech, CO., LTD, China], according to the manufacturer’s instructions.

### Measurement of superoxide dismutase (SOD) and malonaldehyde (MDA) levels

SOD (A001-1) and MDA (A003-1) kits were purchased from Jiancheng Bioengineering Institute (Nanjing, China). The HK2 cells were collected to measure the levels of SOD (hydroxylamine method) and MDA (thiobarbituric acid method) following the manufacturer’s instructions.

### Cell viability assays

Cell viability was assayed using Cell Counting Kit-8 (CCK-8), which is based on the conversion of an orange-colored product from water-soluble tetrazolium salt by dehydrogenases in live cells. CCK-8 (YeSen, China) was used to measure the cell viability of HK2 cells and fibroblasts under different treatment conditions according to the manufacturer’s instructions.

### Iron analysis

A total of 10 mg renal tissues for each group were cut and washed with cold PBS, and then homogenized by vibrating homogenizer immediately. For HK2 cells, the collected cells were homogenized by the ultrasonic cell disrupter. The relative iron concentrations in renal tissues and HK2 cell lysates were assessed using an iron assay kit (A039-2-1, Jiancheng Bioengineering Institute, Nanjing, China) according to the manufacturer’s instructions.

### Statistical analysis

All data are expressed as mean ± standard deviation (SD). Comparisons between two groups were analyzed with an unpaired Student’s *t*-test. Comparisons among different groups were performed using one-way analysis of variance followed by the Student–Newman–Keuls test. Statistical significance was set at *P* < 0.05. All analyses were performed using GraphPad Prism 6.0.

## Results

### Lip-1 alleviated UUO-induced ferroptosis of TECs in kidney tissues

**Fig. 1:**
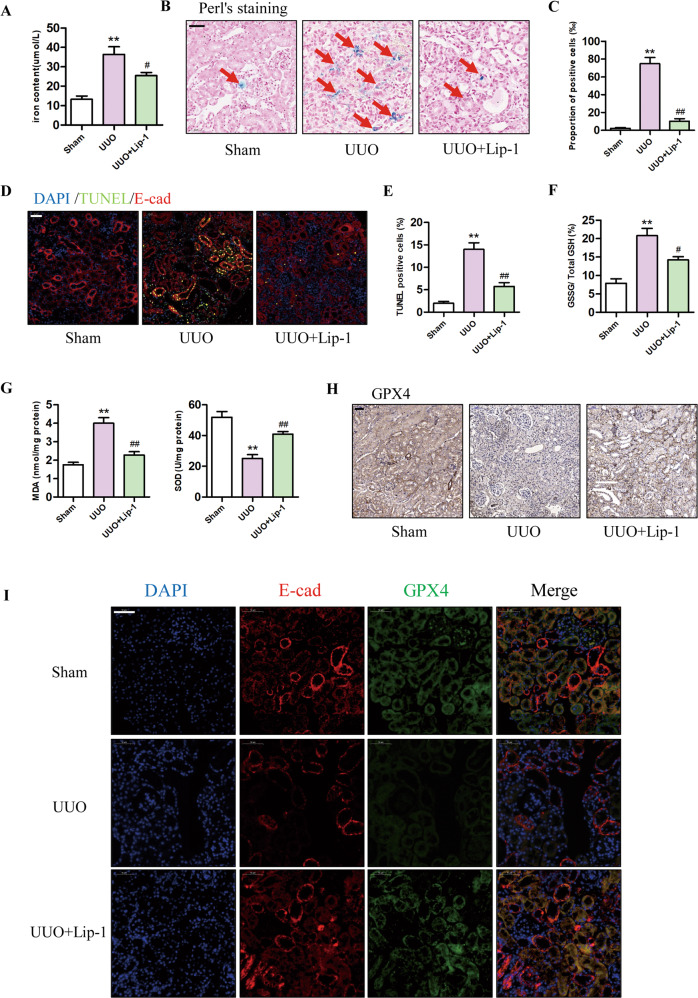
Effects of Liproxstatin-1 on renal iron deposition, cell death, lipid peroxidation, and GPX4 expression in the UUO model. **A** Iron concentrations in the kidney tissue lysates (*n* = 4, **P < 0.01 vs Sham group; ^#^*P* < 0.05 vs UUO group). **B** Perl’s staining of kidney tissues (bar = 50 μm). **C** Proportion of Perl’s staining positive cells (*n* = 4, **P < 0.01 vs Sham group; ^##^*P* < 0.01 vs UUO group). **D** E-cad and TUNEL co-staining in the kidney tissues, DAPI (blue), E-cad (red), and TUNEL (green) (bar = 50 μm). **e** Quantification of TUNEL-positive cells in the kidney tissues (*n* = 4, **P < 0.01 vs Sham group; ^##^*P* < 0.01 vs UUO group). **F** GSSG/total GSH ratio in kidney tissues (*n* = 4, **P < 0.01 vs Sham group; ^#^*P* < 0.05 vs UUO group). **G** MDA and SOD levels in the kidney tissues (*n* = 4, **P < 0.01 vs Sham group; ^##^*P* < 0.01 vs UUO group). **H** Immunohistochemical staining results of GPX4 in kidney tissues (bar = 50 μm). **I** Immunofluorescent analysis with nuclear stained with DAPI (blue), E-cad (red), and GPX4 (green) in kidney tissue sections (bar = 50 μm). The data are presented as the mean ± SD.

Ferroptosis is characterized by the accumulation of lipid peroxidation products, which require abundant levels of iron. Therefore, we first investigated the iron content in renal tissues. As shown in Fig. 1A, B, renal iron concentrations were significantly higher in UUO mice compared with those in the sham group, and treatment with 10 mg/kg Lip-1 reduced the levels of renal iron in the UUO mice. In addition, we detected iron-positive cells in the renal tissues and found Lip-1 treatment significantly reduced the number of iron-positive cells in the UUO + Lip-1 group compared with the UUO group (Fig. 1C). TUNEL staining showed an increased number of cell death of proximal tubular epithelia cells in the UUO group compared with that in the sham group. Lip-1 treatment reduced the number of TUNEL-positive cells in the UUO + Lip-1 group compared with the UUO group (Fig. 1D, E). We then assessed whether Lip-1 inhibited GSSG/ GSH ratio and ROS production in UUO mice. As shown in Fig. 1F, GSSG/ GSH ratio was significantly higher in the UUO group compared with that in the sham group and the Lip-1 treatment significantly decreased the ratio in the UUO group. MDA content was significantly higher in the UUO group compared with that in the sham group, while Lip-1 decreased the levels of MDA in the UUO group. Moreover, SOD levels in the UUO group were markedly lower compared with those in the sham group, and Lip-1 treatment inhibited the downregulation of SOD expression (Fig. 1G). GPX4 is the central regulator of ferroptosis and a deficiency in GPX4 activity is often used as a marker of ferroptosis [19, 20]. In the current study, we evaluated GPX4 expression in kidneys using immunohistochemical and immunofluorescence staining (Fig. 1H, I). The immunohistochemical staining results showed that GPX4 expression was markedly decreased in the UUO group compared with that in the sham group. Intervention with Lip-1 treatment significantly inhibited the downregulation of GPX4 expression in UUO mice. To determine whether the reduction of GPX4 levels occurred mainly in renal TECs, the expression of E-cadherin (epithelial cell marker) and GPX4 were evaluated in kidney tissues using immunofluorescence staining. We found that the level of GPX4 in epithelial cells (E-cadherin positive) was significantly lower in the UUO group compared with that in the sham group. Compared with that in the UUO group, GPX4 levels after Lip-1 treatment were upregulated in the UUO + Lip-1 group. These data revealed that ferroptosis occurred in TECs during UUO-induced renal fibrosis and that Lip-1 was able to inhibit the ferroptosis.

### Lip-1 mitigated morphological changes and renal function impairment in the UUO model

**Fig. 2:**
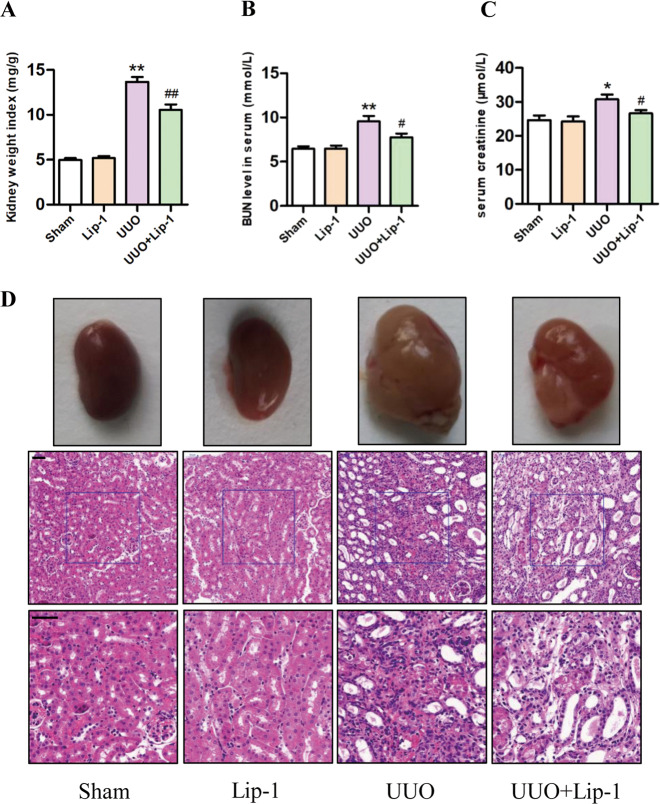
Effects of Liproxstatin-1 on kidney weight index, renal function, and histological alteration in the UUO model. **A** Ratio of obstructed kidney/body weight in mice (*n* = 6, **P < 0.01 vs Sham group; ^##^*P* < 0.01 vs UUO group). **B**, **C** Measurement of the levels of BUN and Scr in serum of mice (*n* = 5, *P < 0.05, **P < 0.01 vs Sham group; ^#^*P* < 0.05 vs UUO group). **D** Morphology of the kidney samples and representative micrographs of H&E staining (bar = 50 μm). The data are presented as the mean ± SD.

As shown in Fig. 2A, the ratio of obstructed kidney weight/body weight (kidney weight index) was higher in the UUO group compared with that in the sham group. However, Lip-1 treatment attenuated the kidney weight index of the UUO group. As shown in Fig. 2B, C, BUN and Scr levels were significantly higher in the UUO group compared with those in the sham group and Lip-1 treatment significantly decreased Scr and BUN levels, indicating an ability of Lip-1 to improve renal function. Histological examination of renal tissues using H&E staining revealed representative features of the obstructive fibrotic response. For instance, the glomerular and tubular structures were normal in the sham and Lip-1 groups, and tubules were closely packed in the renal tissue, indicating that Lip-1 treatment (10 mg/kg/day) caused no obvious damage to the renal structure. In the UUO group, interstitial spaces were significantly wider with the infiltration of inflammatory cells and perivascular exudates, while glomerular capillaries became dilated. In the UUO + Lip-1 group, the morphological changes induced by UUO were significantly improved (Fig. 2D). These results showed that Lip-1 was able to prevent the morphological changes and renal function impairment that were induced by UUO in vivo.

### Lip-1 alleviated renal collagen deposition in the UUO model

**Fig. 3:**
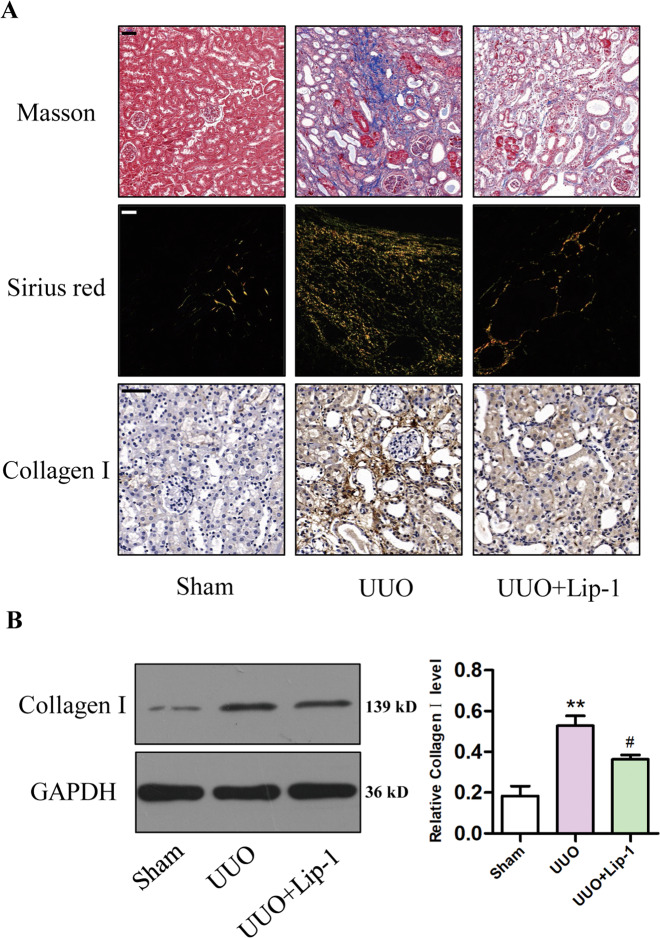
Effects of Liproxstatin-1 on collagen deposition in the UUO model. **A** Renal fibrosis was evaluated by Masson’s trichrome staining (bar = 50 μm), Sirius red staining (bar = 50 μm), and immunohistochemical staining of collagen I (bar = 50 μm). **B** Protein expression of collagen I in renal tissues of mice detected by western blotting (*n* = 3, **P < 0.01 vs sham group; ^#^*P* < 0.05 vs UUO group). The data are presented as the mean ± SD.

Collagen deposition is a significant characteristic of renal fibrosis. As shown in Fig. 3A, collagen deposition stained with Masson’s trichrome and Sirius red was not observed in the sham or Lip-1 groups, whereas it was obvious in the UUO group. Compared with that of the UUO group, Lip-1-treated mice showed reduced collagen deposition in renal tissue. Moreover, immunohistochemical staining revealed the expression of collagen I was markedly increased in renal tissues from the UUO group and significantly reduced in the UUO + Lip-1 group (Fig. 3A). Furthermore, western blot analysis showed that the protein expression of collagen I was significantly higher in UUO mice compared with that in the sham group. Intervention with Lip-1 significantly inhibited the upregulation of collagen I expression (Fig. 3B). These data demonstrated that Lip-1 attenuated UUO-induced renal interstitial collagen deposition.

### Lip-1 reduced secretion of profibrotic factors and myofibroblast activation in the UUO model

**Fig. 4:**
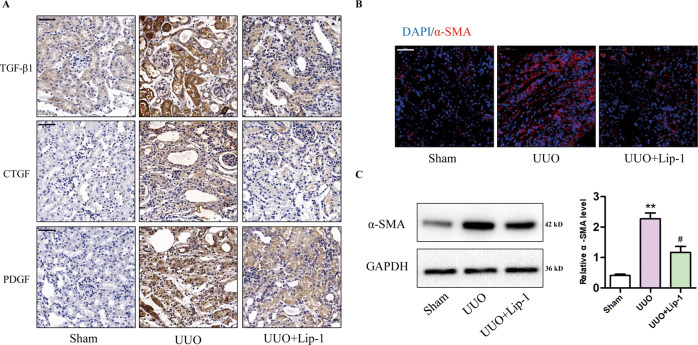
Effects of Liproxstatin-1 on profibrotic factor secretion and myofibroblast activation in the UUO model. **A** Immunohistochemical staining of TGF-β1, CTGF, and PDGF (bar = 50 μm). **B** Immunofluorescent analysis with nuclear stained with DAPI (blue), ɑ-SMA (red) in kidney tissue sections (bar = 50 μm). **C** Protein expression of ɑ-SMA in renal tissues of mice detected by western blotting (*n* = 3, **P < 0.01 vs sham group; ^#^*P* < 0.05 vs UUO group). The data are presented as the mean ± SD.

Profibrotic factors, such as TGF-β1, CTGF, and PDGF, have been implicated in the progression of kidney disease. To investigate how Lip-1 alleviated renal fibrosis, we evaluated protein expression levels of profibrotic factors TGF-β1, CTGF, and PDGF. Immunohistochemical staining showed the expression of TGF-β1, CTGF, and PDGF were markedly increased in renal tissues of the UUO group compared with that in the sham group. Lip-1 treatment significantly reduced TGF-β1, CTGF, and PDGF expression in the UUO + Lip-1 group compared with that in the UUO group (Fig. 4A). To determine whether Lip-1 was able to mitigate myofibroblast activation, we used immunofluorescence staining and western blot analyses to evaluate the expression of ɑ-SMA, which is a major molecular marker of myofibroblasts. Immunofluorescence staining showed that the number of α-SMA-positive cells significantly increased in the UUO group compared with that in the sham group. Lip-1 treatment reduced the number of α-SMA-positive cells in the UUO + Lip-1 group compared with that in the UUO group (Fig. 4B). Furthermore, western blot analysis confirmed that the expression of α-SMA in renal tissue of the UUO + Lip-1 group was significantly lower compared with that in renal tissue of the UUO group (Fig. 4C). These results revealed Lip-1 was able to mediate the secretion of profibrotic factors and myofibroblast activation in kidney tissues that were induced by UUO.

### Lip-1 inhibited ferroptosis and secretion of profibrotic factors in HK2 cells

**Fig. 5:**
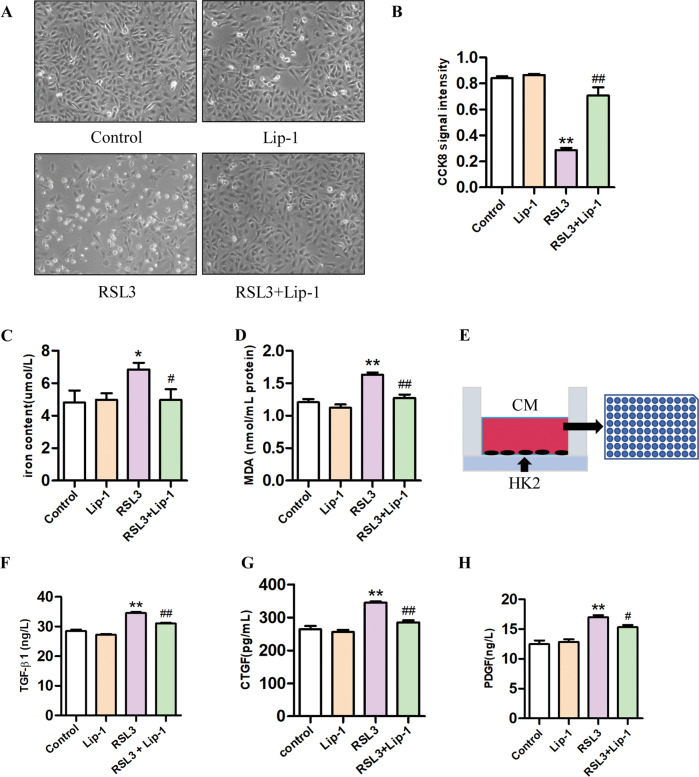
Effects of Liproxstatin-1 on RSL3-induced HK2 cells ferroptosis and profibrotic factor secretion. **A** The morphological of HK2 cells by a light microscope after the treatment for 24 h. **B** Viability of HK2 cells, evaluated by the CCK-8 assay (*n* = 3, **P < 0.01 vs control group; ^##^*P* < 0.01 vs RSL3 group). **C** Iron concentrations in HK2 cells (*n* = 6, *P < 0.05 vs control group; ^#^*P* < 0.05 vs RSL3 group). **D** MDA levels in the HK2 cells (*n* = 6, **P < 0.01 vs control group; ^##^*P* < 0.01 vs RSL3 group). **E** Experimental design. **F**–**H** TGF-β1, CTGF, and PDGF levels in the HK2 cells conditioned medium (*n* = 3, **P < 0.01 vs control group; ^#^*P* < 0.05, ^##^*P* < 0.01 vs RSL3 group). The data are presented as the mean ± SD.

**Fig. 6:**
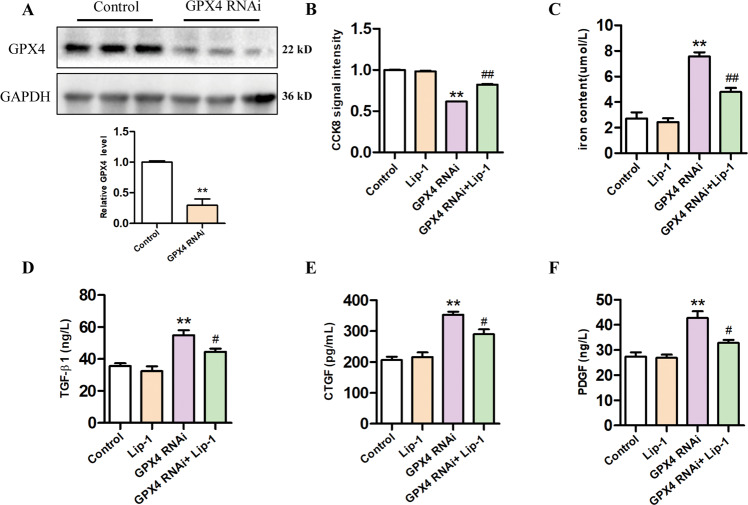
Effects of Liproxstatin-1 on GPX4 knockdown-induced HK2 cells ferroptosis and profibrotic factor secretion. **A** Protein expression of GPX4 in renal tissues of mice was detected by western blotting (*n* = 3, **P < 0.01 vs control group). **B** Viability of HK2 cells, evaluated by the CCK-8 assay (*n* = 5, **P < 0.01 vs control group; ^##^*P* < 0.01 vs GPX4 RNAi group). **c** Iron concentrations in HK2 cells (*n* = 3, **P < 0.01 vs control group; ^##^*P* < 0.01 vs GPX4 RNAi group). **D**–**F** TGF-β1, CTGF, and PDGF levels in the HK2 cells supernatant (*n* = 3, **P < 0.01 vs control group; ^#^*P* < 0.05 vs GPX4 RNAi group). The data are presented as the mean ± SD.

Animal experiments have shown that levels of profibrotic factors increase during the process of renal fibrosis; however, their source is not known. Therefore, we investigated whether profibrotic factors would be released when HK2 cells underwent ferroptosis. Ferroptosis can be induced by pharmacological inhibition of GPX4 (e.g., with RSL3), or via inducible deletion of the gene encoding GPX4 [21]. In this study, we used RSL3 treatment or knockdown of GPX4 level by RNAi in HK2 cells to induce ferroptosis. The CCK-8 assays showed that HK2 cells treated with 0.5 μM Lip-1 exhibited no obvious deleterious effects on proliferation, while treatment with the ferroptosis activator RSL3 (1.0 μM) significantly decreased cell viability. Furthermore, Lip-1 protected HK2 cells from RSL3-induced injury (Fig. 5A, B). Our results also showed that iron concentrations were significantly higher in the RSL3-treated group compared with that in the control group, and Lip-1 treatment reduced the iron content in the cells (Fig. 5C). Furthermore, we found that Lip-1 decreased the RSL3-induced expression of MDA (Fig. 5D). Then, we used ELISA analysis to determine the levels of TGF-β, CTGF, and PDGF in the HK2-CM (Fig. 5E). As shown in Fig. 5F–H, RSL3 increased the level of TGF-β, CTGF, and PDGF in HK2-CM, while the levels were significantly reduced in the RSL3 + Lip-1 group. In addition, we knocked down the GPX4 level in HK2 cells by RNAi. We first examined the protein expression of GPX4 in cells by western blot and confirmed the successful knockdown of GPX4 (Fig. 6A). The CCK-8 assays showed that knockdown of GPX4 significantly decreased cell viability, Lip-1 remarkably extended survival compared with the GPX4 RNAi group (Fig. 6B). Next, we tested the iron content in the cells and the content of TGF-β, CTGF, and PDGF in the supernatant. Lip-1 remarkably reduced the iron content in the cells and secretion of profibrotic factors in HK2 cells (Fig. 6C–F). These findings indicated HK2 cells secreted various profibrotic factors during ferroptosis and that Lip-1 was able to inhibit ferroptosis and thereby inhibit the secretion of the profibrotic factors during the process.

### Profibrotic factors secretion during HK2 cells ferroptosis were involved in fibroblast activation and proliferation

**Fig. 7:**
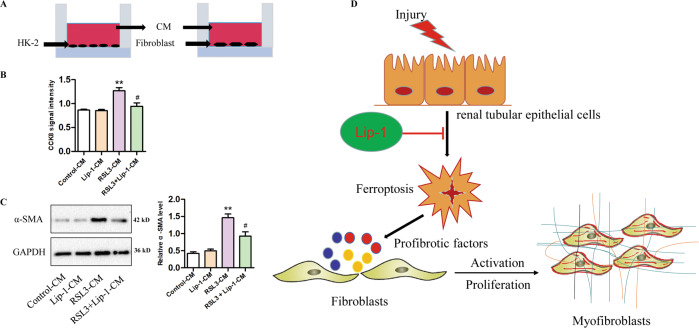
Effects of HK2 cells conditioned medium on fibroblast activation and proliferation. **A** Experimental design. **B** Viability of fibroblast, evaluated by the CCK-8 assay (*n* = 3, **P < 0.01 vs control-CM group; ^#^*P* < 0.05 vs RSL3-CM group). **C** Protein expression of α-SMA in fibroblast, determined by western blotting (*n* = 3, **P < 0.01 vs control-CM group; ^#^*P* < 0.05 vs RSL3-CM group). **D** Hypothetical model showing the role of renal tubular epithelial cells ferroptosis in renal fibrosis and the effect of Liproxstatin-1. The data are presented as the mean ± SD.

To investigate the interactions between TECs and surrounding fibroblasts, we collected RSL3-induced HK2-CM and co-cultured with fibroblasts (Fig. 7A). CCK-8 result showed that the CM of the control group and Lip-1 group have no effect on fibroblast proliferation. CM from RSL3 significantly increased fibroblast cell proliferation, while the proliferation was significantly reduced in the RSL3 + Lip-1 CM group (Fig. 7B). Furthermore, we detected the expression level of α-SMA using western blot analysis. Our findings indicated that CM from RSL3-treated HK2 cells significantly enhanced the expression of α-SMA in fibroblasts, but its expression was inhibited in the RSL3 + Lip-1-CM group (Fig. 7C). These findings suggest that profibrotic factors, including TGF-β, CTGF, and PDGF, released by HK2 cells during ferroptosis could promote fibroblast proliferation, as well as fibroblast-to-myofibroblast differentiation. Furthermore, these effects could be inhibited by treatment with Lip-1.

## Discussion

Increasing evidence shows that TECs are a driving force in the progression of CKD with fibroblasts and myofibroblasts being the final executors of renal fibrosis [4, 6, 22]. Upon injury, TECs give rise to a change in the microenvironment of the tubulointerstitial space in which myofibroblasts are activated [23]. TECs act as the initial reactors after injury, while myofibroblasts act as the final executors. Recent studies have shown that there are five major sources of myofibroblasts, including resident fibroblasts, TECs, endothelial cells, pericytes, and bone marrow-derived fibrocytes [24]. Myofibroblasts are the activated form of fibroblasts and are characterized by ɑ-SMA expression, cell proliferation, and matrix production [25]. However, the exact contribution of each myofibroblast origin to renal fibrosis remains unclear. Despite differing origins, it is commonly viewed that myofibroblasts are mainly activated through a paracrine manner via a variety of cytokines and growth factors produced by TECs, including TGF-β1, Wnt ligands, PDGF, hedgehog, CTGF, hepatocyte growth factor, and angiotensin II [26]. Thus, understanding the crosstalk between myofibroblasts and TECs is of great importance.

Apoptosis and necrosis have been well recognized as the main forms of cell death; however, an increasing body of evidence has revealed that non-apoptotic cell death is a genetically regulated class cell death called “regulated necrosis” [27]. Ferroptosis is a newly described form of regulated necrosis that is characterized by an increase in detrimental lipid ROS produced via iron-dependent lipid peroxidation [28, 29]. Compelling evidence shows that ferroptosis positively affects inflammation and some ferroptosis inhibitors have been shown to exert anti-inflammatory activity in experimental models of certain diseases [30]. However, whether profibrotic factors are secreted during ferroptosis remains unclear. Recently, ferroptosis has been shown to be involved in multiple pathological conditions, including acute renal injury. But it has not been shown whether ferroptosis is induced during renal fibrosis and its potential role in renal fibrosis has not been studied. Repeated and severe episodes of acute kidney injury are recognized as major risk factors for the development of CKD and end-stage kidney disease (ESRD) [31, 32]. One of the important findings obtained in the current study was that ureteral obstruction was able to induce ferroptosis in renal TECs in vivo. Furthermore, ferroptosis-mediated tubular cell death led to the secretion of various profibrotic factors, which could then affect interstitial fibroblasts in a paracrine fashion to accelerate the proliferation and differentiation of these cells.

The appearance of ferroptosis helps explain many mechanisms regarding drug action and disease. Interfering with the progress of ferroptosis is a promising approach for treating certain diseases [33]. Lip-1 is a spiroquinoxalinamine derivative and a potent inhibitor of ferroptosis. However, the mechanism by which Lip-1 protects against ferroptosis is yet to be elucidated. The results of the current study demonstrated Lip-1 was a safe and effective drug that showed an antifibrotic effect and improved histopathological abnormalities in mice of the UUO model. Our results showed that the antifibrotic effect of Lip-1 might be due to the inhibition of ferroptosis in TECs. In addition, we found that Lip-1 can attenuate the expression of profibrotic factors in the UUO model and ferroptosis of HK2 cells. In order to prove that the effect of Lip-1 on the proliferation and differentiation of fibroblasts, we tested the expression of profibrotic factors in the HK2 cells culture medium. We found that Lip-1 can reduce the expression of TGF-β1, CTGF, and PDGF in HK2-CM. In addition, the co-culture of RSL3-induced CM and fibroblasts proved the profibrotic factors secretion during ferroptosis is involved in fibroblast proliferation and activation, and this effect can be inhibited by Lip-1.

In summary, we provided new mechanistic insight into renal fibrosis and determined that Lip-1 attenuated UUO-induced kidney fibrosis by inhibiting ferroptosis-mediated cell death. Lip-1 was able to reduce the activation and proliferation of surrounding fibroblasts by inhibiting the paracrine activity of profibrotic factors secreted by epithelial cells. However, this study has several limitations. First, we were not able to directly determine whether the proliferation of fibroblasts in vivo was caused by the ferroptosis of epithelial cells. Second, various strategies may be useful to assess the direct relationship between ferroptosis and renal fibrosis in the future, including the use of *GPX4* knockout animals and *GPX4* knockout cells. Third, we did not use other ferroptosis inhibitors and explore their roles in renal fibrosis. Finally, the specific mechanism involved in the secretion of profibrotic factors during ferroptosis of renal TECs remains unclear. These limitations should be addressed in future studies.

In conclusion, our study found that ferroptosis played an important role in UUO-induced renal fibrosis and the ferroptosis inhibitor Lip-1 attenuated UUO-induced kidney fibrosis by inhibiting ferroptosis-mediated TECs death. In addition, Lip-1 could reduce the activation of surrounding fibroblasts by inhibiting the paracrine activity of profibrotic factors of epithelial cells (Fig. 7D). The ferroptosis inhibitor Lip-1 may be an effective therapy for kidney fibrosis.

## Data Availability

The data sets used and/or analyzed during the current study are available from the corresponding author on reasonable request.

## References

[1] Lu X, Rudemiller NP, Ren J, Wen Y, Yang B, Griffiths R (2019). Opposing actions of renal tubular- and myeloid-derived porcupine in obstruction-induced kidney fibrosis. Kidney Int.

[2] Humphreys BD (2018). Mechanisms of renal fibrosis. Annu Rev Physiol.

[3] Martinez-Klimova E, Aparicio-Trejo OE, Tapia E, Pedraza-Chaverri J. Unilateral ureteral obstruction as a model to investigate fibrosis-attenuating treatments. Biomolecules. 2019;9:141. 10.3390/biom9040141 PMC6523883 30965656

[4] Liu BC, Tang TT, Lv LL, Lan HY (2018). Renal tubule injury: a driving force toward chronic kidney disease. Kidney Int.

[5] Hosseinian S, Rad AK, Bideskan AE, Soukhtanloo M, Sadeghnia H, Shafei MN (2017). Thymoquinone ameliorates renal damage in unilateral ureteral obstruction in rats. Pharm Rep.

[6] Canaud G, Brooks CR, Kishi S, Taguchi K, Nishimura K, Magassa S, et al. Cyclin G1 and TASCC regulate kidney epithelial cell G2-M arrest and fibrotic maladaptive repair. Sci Transl Med. 2019;11:eaav4754. 10.1126/scitranslmed.aav4754 PMC6527117 30674655

[7] Grande MT, Sanchez-Laorden B, Lopez-Blau C, De Frutos CA, Boutet A, Arevalo M (2015). Snail1-induced partial epithelial-to-mesenchymal transition drives renal fibrosis in mice and can be targeted to reverse established disease. Nat Med.

[8] Li H, Shao F, Qian B, Sun Y, Huang Z, Ding Z (2019). Upregulation of HER2 in tubular epithelial cell drives fibroblast activation and renal fibrosis. Kidney Int.

[9] Dixon SJ, Lemberg KM, Lamprecht MR, Skouta R, Zaitsev EM, Gleason CE (2012). Ferroptosis: an iron-dependent form of nonapoptotic cell death. Cell.

[10] Wenzel SE, Tyurina YY, Zhao J, St Croix CM, Dar HH, Mao G (2017). PEBP1 wardens ferroptosis by enabling lipoxygenase generation of lipid death signals. Cell.

[11] Zhu H, Sun A (2018). Programmed necrosis in heart disease: molecular mechanisms and clinical implications. J Mol Cell Cardiol.

[12] Deng F, Sharma I, Dai Y, Yang M, Kanwar YS (2019). Myo-inositol oxygenase expression profile modulates pathogenic ferroptosis in the renal proximal tubule. J Clin Invest.

[13] Sun X, Niu X, Chen R, He W, Chen D, Kang R (2016). Metallothionein-1G facilitates sorafenib resistance through inhibition of ferroptosis. Hepatology.

[14] Friedmann Angeli JP, Schneider M, Proneth B, Tyurina YY, Tyurin VA, Hammond VJ (2014). Inactivation of the ferroptosis regulator Gpx4 triggers acute renal failure in mice. Nat Cell Biol.

[15] Linkermann A, Chen G, Dong G, Kunzendorf U, Krautwald S, Dong Z (2014). Regulated cell death in AKI. J Am Soc Nephrol.

[16] Feng Y, Madungwe NB, Imam Aliagan AD, Tombo N, Bopassa JC (2019). Liproxstatin-1 protects the mouse myocardium against ischemia/reperfusion injury by decreasing VDAC1 levels and restoring GPX4 levels. Biochem Biophys Res Commun.

[17] Chen X, Zhang B, Liu T, Feng M, Zhang Y, Zhang C (2019). Liproxstatin-1 attenuates morphine tolerance through inhibiting spinal ferroptosis-like cell death. ACS Chem Neurosci.

[18] Zhang B, Liu P, Zhou Y, Chen Z, He Y, Mo M (2019). Dihydroartemisinin attenuates renal fibrosis through regulation of fibroblast proliferation and differentiation. Life Sci.

[19] Yang WS, SriRamaratnam R, Welsch ME, Shimada K, Skouta R, Viswanathan VS (2014). Regulation of ferroptotic cancer cell death by GPX4. Cell.

[20] Conrad M, Proneth B (2020). Selenium: tracing another essential element of ferroptotic cell death. Cell Chem Biol.

[21] Shah R, Shchepinov MS, Pratt DA (2018). Resolving the role of lipoxygenases in the initiation and execution of ferroptosis. ACS Cent Sci.

[22] Kuppe C, Ibrahim MM, Kranz J, Zhang X, Ziegler S, Perales-Paton J (2021). Decoding myofibroblast origins in human kidney fibrosis. Nature.

[23] Hong W, Zhang G, Lu H, Guo Y, Zheng S, Zhu H (2019). Epithelial and interstitial Notch1 activity contributes to the myofibroblastic phenotype and fibrosis. Cell Commun Signal.

[24] LeBleu VS, Taduri G, O’Connell J, Teng Y, Cooke VG, Woda C (2013). Origin and function of myofibroblasts in kidney fibrosis. Nat Med.

[25] Hinz B, Lagares D (2020). Evasion of apoptosis by myofibroblasts: a hallmark of fibrotic diseases. Nat Rev Rheumatol.

[26] Qi R, Yang C (2018). Renal tubular epithelial cells: the neglected mediator of tubulointerstitial fibrosis after injury. Cell Death Dis.

[27] Lu LQ, Tian J, Luo XJ, Peng J (2021). Targeting the pathways of regulated necrosis: a potential strategy for alleviation of cardio-cerebrovascular injury. Cell Mol Life Sci.

[28] Minagawa S, Yoshida M, Araya J, Hara H, Imai H, Kuwano K (2020). Regulated necrosis in pulmonary disease. a focus on necroptosis and ferroptosis. Am J Respir Cell Mol Biol.

[29] Mou Y, Wang J, Wu J, He D, Zhang C, Duan C (2019). Ferroptosis, a new form of cell death: opportunities and challenges in cancer. J Hematol Oncol.

[30] Sun Y, Chen P, Zhai B, Zhang M, Xiang Y, Fang J (2020). The emerging role of ferroptosis in inflammation. Biomed Pharmacother.

[31] He L, Wei Q, Liu J, Yi M, Liu Y, Liu H (2017). AKI on CKD: heightened injury, suppressed repair, and the underlying mechanisms. Kidney Int.

[32] Kumar S (2018). Cellular and molecular pathways of renal repair after acute kidney injury. Kidney Int.

[33] Qiu Y, Cao Y, Cao W, Jia Y, Lu N (2020). The application of ferroptosis in diseases. Pharm Res.

